# Genome-wide screen for modifiers of *Na*^*+*^*/K*^*+*^*ATPase* alleles identifies critical genetic loci

**DOI:** 10.1186/s13041-014-0089-3

**Published:** 2014-12-05

**Authors:** Aaron D Talsma, John F Chaves, Alexandra LaMonaca, Emily D Wieczorek, Michael J Palladino

**Affiliations:** Department of Pharmacology & Chemical Biology, University of Pittsburgh School of Medicine, 3501 Fifth Avenue, BST3 7042, Pittsburgh, PA 15261 USA; Pittsburgh Institute for Neurodegenerative Diseases, University of Pittsburgh School of Medicine, 3501 Fifth Avenue, BST3 7042, Pittsburgh, PA 15261 USA

**Keywords:** *Drosophila melanogaster*, ATPalpha, Sodium pump, Temperature-sensitive paralysis, Conditional paralysis, Seizure, Migrane, Screen, Genome-wide, Seizure suppressor

## Abstract

**Background:**

Mutations affecting the *Na*^*+*^*/ K*^*+*^*ATPase* (a.k.a. the sodium-potassium pump) genes cause conditional locomotor phenotypes in flies and three distinct complex neurological diseases in humans. More than 50 mutations have been identified affecting the human *ATP1A2* and *ATP1A3* genes that are known to cause rapid-onset Dystonia Parkinsonism, familial hemiplegic migraine, alternating hemiplegia of childhood, and variants of familial hemiplegic migraine with neurological complications including seizures and various mood disorders. In flies, mutations affecting the *ATPalpha* gene have dramatic phenotypes including altered longevity, neural dysfunction, neurodegeneration, myodegeneration, and striking locomotor impairment. Locomotor defects can manifest as conditional bang-sensitive (BS) or temperature-sensitive (TS) paralysis: phenotypes well-suited for genetic screening.

**Results:**

We performed a genome-wide deficiency screen using three distinct missense alleles of *ATPalpha* and conditional locomotor function assays to identify novel modifier loci. A secondary screen confirmed allele-specificity of the interactions and many of the interactions were mapped to single genes and subsequently validated. We successfully identified 64 modifier loci and used classical mutations and RNAi to confirm 50 single gene interactions. The genes identified include those with known function, several with unknown function or that were otherwise uncharacterized, and many loci with no described association with locomotor or Na^+^/K^+^ ATPase function.

**Conclusions:**

We used an unbiased genome-wide screen to find regions of the genome containing elements important for genetic modulation of ATPalpha dysfunction. We have identified many critical regions and narrowed several of these to single genes. These data demonstrate there are many loci capable of modifying ATPalpha dysfunction, which may provide the basis for modifying migraine, locomotor and seizure dysfunction in animals.

**Electronic supplementary material:**

The online version of this article (doi:10.1186/s13041-014-0089-3) contains supplementary material, which is available to authorized users.

## Background

In many organisms, highly conserved Na^+^/K^+^ ATPases are responsible for maintaining ion gradients across the plasma membrane through ATP-dependent asymmetric translocation of Na^+^ and K^+^ ions. These ion gradients maintain the resting potential of cells, which facilitates neural signaling and many essential secondary processes. Mature Na^+^/K^+^ ATPase complexes are heteromultimers of alpha, beta, and gamma subunits in mammals. Flies express only the alpha and beta subunits, the former of which is known as ATPalpha. Like its mammalian homologue, ATPalpha contains ten transmembrane domains and has the ATP-dependent catalytic activity essential for pump function [1-3].

Mutations affecting the alpha subunit of the Na^+^/K^+^ ATPase in humans are associated with at least three human diseases: Rapid-onset Dystonia Parkinsonism (RDP), Familial Hemiplegic Migraine (FHM), and Alternating Hemiplegia of Childhood (AHC; [4]). RDP is a severe DOPA non-responsive form of dystonia the etiology of which is poorly understood [5]. FHM, possibly the most severe form of migraine, is associated with a debilitating partial paralysis, and currently is largely untreatable [6]. AHC is a severe childhood locomotor disease associated with recurring acute bouts of paralysis and muscle weakness, and general developmental delays (reviewed by [7]). Recently Sasaki and colleagues have described several children who seem to have a disease intermediate to AHC and RDP [8]. All of these diseases are complex neuromuscular conditions associated with marked locomotor dysfunction and for which the underlying pathogenesis is poorly understood.

*Drosophila* conditional mutants have been isolated based upon temperature-sensitive (TS) or bang-sensitive (BS) paralysis phenotypes over the past many decades. TS mutants generally become paralyzed in less than five minutes at 38°C and BS mutants paralyze in response to 20 seconds of mechanical stress. These classes of mutants have proven informative and have defined many essential components of neural signaling [9-15]. Conditional TS mutations typically affect critical neural proteins and include well-studied genes such as *para* (voltage-dependent NaCH), *NapTS* (RNA helicase affecting *para* transcripts), *cacophony* (a voltage-gated calcium channel), *ATPalpha* (Na^+^/K^+^ ATPase), *comatose* (dNSF1), *shibire* (Dynamin), *syntaxin*, *synaptobrevin*, and *dao* (regulator of Erg-type K-channels), to name a few [15-24]. Conditional BS mutations can also affect important neural signaling and ion homeostasis proteins, such as para and ATPalpha [23,25]. They also affect many proteins with integral roles in bioenergetics and mitochondrial function, such as sesB, ATP6, kdn, eas, and SOD2 [26-30]. Interestingly, numerous BS mutants have been shown to exhibit seizures and model epilepsies (e.g. *para*^*BSS1*^*, ATP6*^*1*^, and *Kazachoc*; [25,31,32]). BS and TS conditional mutants have proven incredibly important to our understanding of neurobiology and previous studies have successfully used them to identify genes that modify these behaviors (e.g. [33-35]). However, there are no reports of genome-wide screens for modifier loci using these behavioral phenotypes in *Drosophila* or studying *ATPalpha* in any model system. This suggests that such an approach could yield novel loci involved in regulating ion homeostasis or neural excitability.

It has previously been shown that mutations in *ATPalpha* result in profound neural and locomotor dysfunction in *Drosophila* [23,36-40]. Hypomorphic *ATPalpha* alleles, such as *ATPalpha*^*2206*^, display BS paralysis and phenocopy injection of the selective Na^+^/K^+^ ATPase inhibitor, ouabain [39]. The *ATPalpha*^*DTS1*^ mutation is a dominant, conditional, gain-of-function, missense mutation [23]. The mutation results in an E982K substitution near the protein’s C-terminus (short isoform numbering). *ATPalpha*^*DTS1*^ heterozygotes exhibit rapid paralysis at 38°C with complete penetrance. This is thought to be a result of conditional neuronal hyperexcitability caused by the mutation [23]. *ATPalpha*^*CJ5*^ and *ATPalpha*^*CJ10*^ are also dominant missense mutations affecting evolutionarily conserved amino acids [36]. However, they each exhibit unique locomotor phenotypes. *ATPalpha*^*CJ5*^ behaves like a loss-of-function allele of *ATPalpha*, exhibiting haploinsufficiency and BS paralysis [36]. *ATPalpha*^*CJ10*^ exhibits BS and progressive TS phenotypes, suggesting this is a loss-of-function allele that exhibits weak gain-of-function features, which are uncovered with age [36]. Thus, *ATPalpha*^*DTS1*^, *ATPalpha*^*CJ5*^, and *ATPalpha*^*CJ10*^ are all dominant, phenotypically well-characterized, and possibly functionally distinct, conditional locomotor mutants. Such alleles are ideally suited for a modifier screen. Using multiple alleles of *ATPalpha* increases the power of the screen and affords the likelihood of identifying allele-specific modifiers. Furthermore, to our knowledge, this is the first report of a genome wide genetic screen in any animal system using three distinct alleles of the same gene in parallel to identify allele-specific interactions.

Deficiency screens have been effectively used for elucidating novel gene interactions in *Drosophila* using various phenotypes [41-43]. Deficiency (*Df*) strains each have a unique deletion of a segment of the genome. Phenotypically screening for genetic interactions between defined point mutations and an individual defined deficiency is an efficient way to identify modifier loci. Using a collection of *Dfs* covering a high percentage of the genome (95-98%), one can identify critical modifier loci anywhere in the genome. This provides an efficient yet powerful and unbiased forward genetic approach. Critical loci can often be narrowed to single genes using smaller deficiencies and single gene disruptions. We have performed such a screen using *ATPalpha*^*DTS1*^, *ATPalpha*^*CJ5*^ and *ATPalpha*^*CJ10*^, identified 64 critical modifier intervals, and successfully confirmed 50 single-gene modifiers, including numerous novel loci of interest. These data suggest the existence of many susceptibility loci capable of modifying migraine, locomotor and seizure dysfunction in animals and provide a rich data set from which new targets for anti-migraine or anti-epileptic drugs could be drawn.

## Results

### Primary genetic modifier screen

To identify new genes that interact with *ATPalpha* we performed a deficiency screen using three characterized alleles: *ATPalpha*^*DTS1*^, *ATPalpha*^*CJ5*^ and *ATPalpha*^*CJ10*^. We used the Bloomington Stock Center deficiency (*Df*) kit that covers approximately 98% of the *Drosophila* genome. All of the 467 *Df* strains we received were tested with at least one *ATPalpha* mutant allele and the vast majority of strains were tested with multiple alleles (see Table 1). Each of the three *ATPalpha* mutants was mated to each *Df* line. F_1_ progeny bearing *ATPalpha*^*DTS1*^ and each deficiency were subjected to TS assays while progeny bearing *ATPalpha*^*CJ5*^ or *ATPalpha*^*CJ10*^ and each *Df* were assayed for BS. The average response for *ATPalpha*^*DTS1*^*, ATPalpha*^*CJ5*^ and *ATPalpha*^*CJ10*^*Df* double mutants was 34.8+/−25.3, 89.9+/−53.6 and 41.5+/−34.8 seconds, respectively (Additional file 1). We used these values to identify putative genetic interactions. *Df(3R)BSC819* contains a deletion of the *ATPalpha* locus and failed to complement each mutant allele, as expected.

**Table 1:** **Primary screen summary**

	***DTS1***	***CJ5***	***CJ10***
**Number tested in primary screen**	386	393	358
**% of Kit tested**	83%	84%	77%
**Avg. Response (Sec.)**	34	88	42
**St. Dev. (Sec.)**	26.4	74.6	46.1
**Normal Range (Sec.)**	20-60	20-190	10-150
**Number selected for verification**	89	69	78
**Screened phenotype**	TS	BS	BS

**Figure 1:**
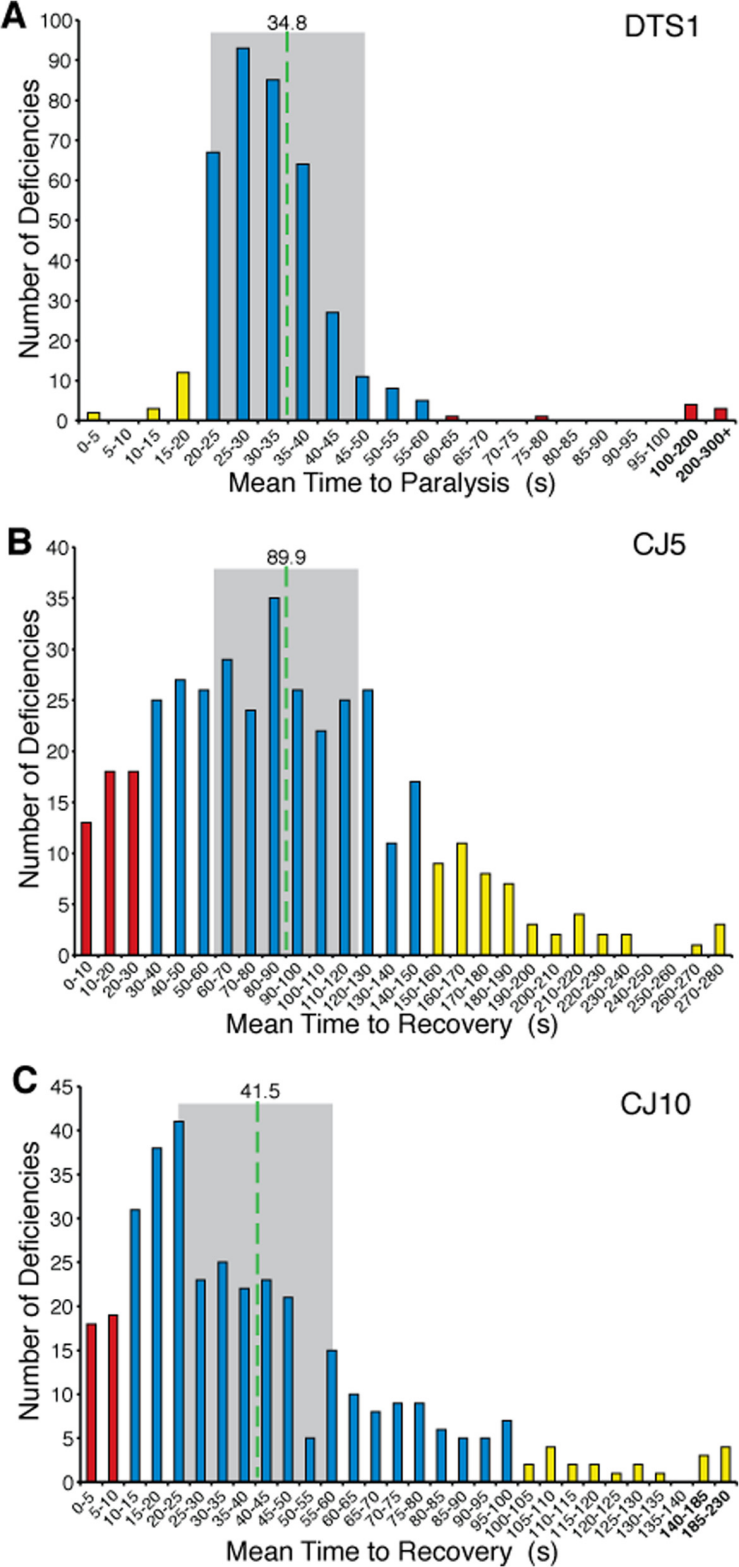
**Distribution of phenotypic modifiers identified through a deficiency screen. A-C)** *ATPalpha* mutant animals also bearing individual unique chromosomal deficiencies (Df) were assayed for conditional locomotor function to identify modifiers. The data reveal a largely normal distribution centered around a typical response (blue) for each mutant. Those deviating from the typical response were termed putative enhancers (yellow) or suppressors (red). **A)** *ATPalpha* ^*CJ5*^ *, Df* double mutants and **B)** *ATPalpha* ^*CJ10*^ *, Df* double mutants were assayed for recovery from mechanical stress at adult day 15. **C)** *ATPalpha* ^*DTS1*^ *, Df* double mutants were assayed for time to TS paralysis on adult day 1. **A-C)** The mean response is shown as a dashed green line. +/− 0.5 Std. Dev. are indicated by gray shading.

**Figure 2:**
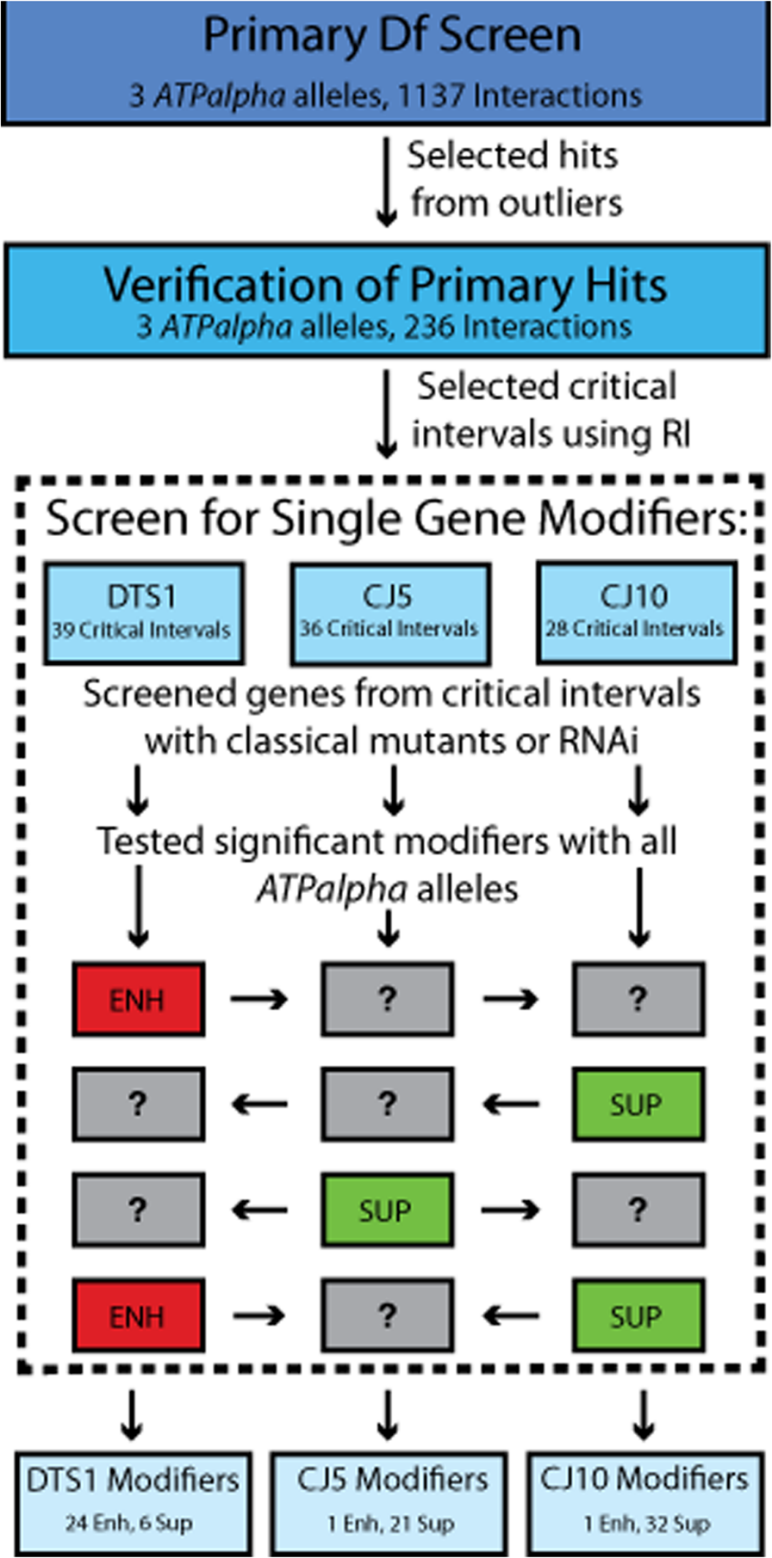
**Schematic of the deficiency screen workflow.** Using the Bloomington deficiency kit, 1137 initial interactions were screened using *ATPalpha* ^*CJ5*^, *ATPalpha* ^*CJ10*^, or *ATPalpha* ^*DTS1*^. Putative enhancers and suppressors were selected for verification with a larger sample size. Any verified interacting deficiencies were deemed critical intervals. Once critical intervals were selected a screen for single gene modifiers from within the intervals was performed using available classical mutants and transgenic RNAi strains. If a modifier was found it was retested with other *ATPalpha* alleles to determine whether the interaction was allele-specific.

The data from the primary screen were organized graphically by average time to recovery or paralysis for each double mutant (Figure 1). In each case, the resulting data formed a largely normal distribution. Double mutants that deviated significantly from the mean were termed putative enhancers or suppressors and were tested again in a verification screen. The workflow for the genetic screen is depicted in Figure 2. In the primary screen, 1137 interactions were examined for the three conditional locomotor mutants identifying 117 putative enhancer, suppressor, or synthetic lethal regions. These interactions were examined further in the verification screen.

### Verification screen

To mitigate the effect of false positives and confirm that interactions were reproducible before pursuing them further, we performed a verification screen (an independent experiment) with the putative modifiers. We began the verification with 89 *ATPalpha*^*DTS1*^ modifiers (Figure 1A), 69 *ATPalpha*^*CJ5*^ modifiers (Figure 1B), and 78 *ATPalpha*^*CJ10*^ modifiers (Figure 1C). After verification, we took advantage of having two data sets (primary and verification screen) and created a formula to determine the reproducibility of each putative genetic interaction (see Materials and Methods). We calculated a reproducibility index (RI) and used it to help us identify the most promising critical intervals. *Dfs* with the highest RIs were prioritized for mapping and secondary screening. This approach yielded 7 putative *ATPalpha*^*DTS1*^ enhancers, 12 suppressors, and five synthetic lethal (enhanced to lethality) combinations (Table 2). The *ATPalpha*^*CJ5*^ screen yielded 13 enhancers, 10 suppressors, and four synthetic lethal combinations (Table 3). The *ATPalpha*^*CJ10*^ primary screen yielded 17 enhancers, 11 suppressors, and one synthetic lethal (Table 4).

**Table 2:** **Confirmed** ***ATPalpha*** ^***DTS1***^ **interacting deficiencies**

**Df Name**	**Enh/Sup**	**Mean +/− SEM**	**Total N**	**RI**	**Hits in region**	**Coincidence**
***DTS1***	***Control***	***37.8 +/− 2.6***	***23***	***-***	***-***	***-***
Df(3 L)Exel6092	Sup	76.4 +/− 33.4	11	6.27	spz5, FMRFaR, scramb2, aly	CJ10
Df(2R)ED1725	Sup	209.8 +/− 57.0	6	4.87		
Df(2R)BSC361	Sup	87.8 +/− 10.6	16	4.68	Stj	CJ10
Df(3 L)BSC33	Sup	103.9 +/− 34.4	14	4.64		
Df(3 L)Exel8104	Enh	27.3 +/− 27.3	11	3.18		
Df(3R)BSC486	Enh	17.2 +/− 1.7	19	2.65		CJ10
Df(2 L)BSC180	Sup	85.3 +/− 16.5	25	2.13	Rbp9	CJ10
Df(3R)Exel6210	Sup	151.3 +/− 42.9	11	2.02		
Df(2R)BSC383	Sup	129.1 +/− 40.9	11	1.80		
Df(2 L)BSC278	Sup	52.3 +/− 14.9	25	1.54		
Df(3 L)BSC23	Enh	11.2 +/− 1.1	14	1.53	spz5, scramb2, rasp, aly	CJ5, CJ10
Df(2 L)Exel6005	Sup	73.9 +/− 23.1	19	1.51		
Df(3R)BSC650	Enh	22.1 +/− 2.3	13	1.20		
Df(2 L)ED1203	Enh	21.2 +/− 2.1	13	0.92	Ham	CJ5
Df(3R)ED2	Enh	20.0 +/− 2.6	25	0.88		
Df(2R)ED3728	Sup	46.3 +/− 8.2	15	0.86		
Df(1)BSC767	Sup	138.2 +/− 26.7	13	0.82		
Df(2R)M60E	Sup	48.1 +/− 5.9	25	0.74	Rpl19, pain	
Df(2 L)ED629	Enh	27.1 +/− 2.9	13	0.49	Glutactin, sema-1a	
Df(3R)ED7665	Enh/Leth	-		-		CJ10
Df(3R)ED6361	Enh/Leth	-		-		
Df(3 L)BSC375	Enh/Leth	-		-		
Df(3R)BSC467	Enh/Leth	-		-		CJ10
Df(1)BSC708	Enh/Leth	-		-		
Df(3R)BSC819	Enh/Leth	-		-	ATPalpha	All Enh/Leth

**Table 3:** **Confirmed** ***ATPalpha*** ^***CJ5***^ **interacting deficiencies**

**Df Name**	**Enh/Sup**	**Mean ± SEM**	**Total N**	**RI**	**Hits in region**	**Coincidence**
***CJ5***	***Control***	***100.0 +/− 11.4***	***28***	***-***	***-***	***-***
Df(3 L)BSC797	Enh	240.6 +/− 17.3	14	4.03		
Df(2 L)BSC214	Enh	178.7 +/− 22.6	15	2.38		
Df(3 L)ED4475	Enh	172.4 +/− 21.4	16	1.97		CJ10
Df(2 L)BSC781	Sup	16.2 +/− 4.2	25	1.93	Cact, CG5888	
Df(3R)BSC547	Enh	165.0 +/− 24.0	17	1.81	Sro, Dop1r2, ppk21	
Df(3 L)M21	Enh	182.5 +/− 26.2	13	1.80		
Df(2R)BSC199	Enh	168.8 +/− 24.2	14	1.63		
Df(3R)ED5495	Enh	182.0 +/− 24.0	16	1.62		
Df(2R)PK1	Sup	26.9 +/− 8.7	20	1.57	Pu	
Df(2 L)Exel6005	Enh	235.9 +/− 22.6	13	1.55		
Df(2 L)H20	Sup	29.2 +/− 6.4	25	1.52		
Df(2 L)ED1203	Sup	31.0 +/− 4.8	23	1.52	ham, ddc	DTS1
Df(3 L)BSC23	Sup	31.6 +/− 8.0	17	1.50	rasp, spz5, scramb2, aly	DTS1, CJ10
Df(2 L)J39	Sup	23.0 +/− 4.7	21	1.43	FKBP59	CJ10
Df(2R)BSC267	Enh	144.7 +/− 24.7	3	1.38		
Df(1)BSC825	Sup	36.7 +/− 7.2	9	1.37		
Df(2 L)BSC213	Enh	146.3 +/− 46.2	8	1.35		
Df(3 L)Exel6112	Enh	143.1 +/− 27.7	14	1.35		CJ10
Df(2 L)ED489	Sup	41.4 +/− 16.2	13	1.28	Ndae1	CJ10
Df(2 L)ED8142	Sup	38.9 +/− 7.9	24	1.20		
Df(2R)BSC429	Sup	40.1 +/− 16.4	16	1.15		
Df(2 L)BSC295	Enh	181.7 +/− 20.2	15	1.09		
Df(2 L)BSC149	Enh	107.1 +/− 41.2	12	1.01		
Df(2 L)BSC233	Enh/Leth	-		-		
Df(3 L)BSC451	Enh/Leth	-		-		
Df(3R)BSC469	Enh/Leth	-		-		CJ10
Df(3R)BSC491	Enh/Leth	-		-		
Df(3R)BSC819	Enh/Leth	-		-	ATPalpha	All Enh/Leth

**Table 4:** **Confirmed** ***ATPalpha*** ^***CJ10***^ **interacting deficiencies**

**Df Name**	**Enh/Sup**	**Mean +/− SEM**	**Total N**	**RI**	**Hits in region**	**Coincidence**
***CJ10***	***Control***	***50.3 +/− 7.1***	***17***	***-***	***-***	***-***
Df(3R)BSC486	Enh	168.5 +/− 38.8	6	4.92		DTS1
Df(3 L)Exel6112	Enh	144.6 +/− 15.4	18	4.20		CJ5
Df(2 L)BSC180	Enh	151.7 +/− 34.5	9	2.93	Rbp9	DTS1
Df(2 L)TW161	Enh	103.1 +/− 18.2	12	2.89		
Df(3R)BSC469	Enh	96.5 +/− 22.9	11	2.59		CJ5
Df(3R)BSC681	Enh	98.7 +/− 49.4	6	2.32		
Df(3R)A113	Enh	92.4 +/− 8.8	14	2.16		
Df(3R)BSC501	Enh	91.8 +/− 7.8	14	2.10	CG14508	
Df(3R)ED5495	Enh	139.6 +/− 34.5	7	1.98		
Df(3 L)Exel6092	Enh	142.8 +/− 31.5	20	1.85	spz5, scramb2, FMRFaR, aly	DTS1
Df(2R)BSC664	Enh	60.2 +/− 14.1	11	1.77		
Df(3R)Exel6196	Enh	109.1 +/− 28.5	11	1.74		
Df(3 L)BSC410	Enh	85.3 +/− 11.1	12	1.54		
Df(3 L)ED4475	Sup	8.0 +/− 1.6	7	1.48		CJ5
Df(3 L)BSC23	Sup	8.0 +/− 3.0	18	1.43	rasp, spz5, scramb2, aly	DTS1, CJ5
Df(2 L)BSC240	Enh	91.8 +/− 10.9	24	1.43	Nckx30C, ppk11, nAChR-alpha6, FKBP59	
Df(2 L)J39	Sup	7.9 +/− 2.2	25	1.43	FKBP59	CJ5
Df(2R)BSC361	Enh	114.0 +/− 26.3	8	1.29	Stj	DTS1
Df(2R)BSC661	Enh	78.0 +/− 10.9	23	1.25		
Df(3R)ED5577	Sup	14.0 +/− 2.0	13	1.20		
Df(2 L)ED489	Sup	12.1 +/− 2.6	25	1.19	Ndae1	CJ5
Df(3 L)ED230	Sup	13.7 +/− 3.7	10	1.17		
Df(4)ED6380	Sup	12.6 +/− 3.6	25	1.14		
Df(3 L)BSC113	Sup	14.3 +/− 1.7	15	1.13	aay	
Df(2 L)ED793	Sup	16.2 +/− 4.1	25	1.09	Dyrk2, NimB5, nAChRα5	
Df(2 L)BSC149	Sup	16.1 +/− 3.3	14	1.09		
Df(3R)ED7665	Sup	16.5 +/− 5.1	21	1.06		DTS1
Df(3 L)BSC442	Enh	79.1 +/− 10.4	15	1.02		
Df(3R)BSC467	Enh/Leth	-		-		DTS1
Df(3R)BSC819	Enh/Leth	-		-	ATPalpha	All Enh/Leth

### Single gene identification and testing

After the verification of critical intervals, genes contained within these intervals were selected for testing. Where practical large intervals were narrowed using smaller Dfs. We obtained classical alleles for integral genes from Bloomington, when possible. Each single gene mutant was mated to the *ATPalpha* allele it putatively modified and to *w*^*1118*^. All single gene mutants displayed no BS or TS phenotype as heterozygotes (data not shown). Heterozygous double mutants were again assayed for TS or BS with age matched controls. Significant interacting single gene mutants were also tested with the other *ATPalpha* alleles (Figure 2). Twenty single gene interactions were found using classical mutants for *ATPalpha*^*DTS1*^ including 19 single gene enhancers and one single gene suppressor. Ten single gene suppressors were found for *ATPalpha*^*CJ5*^. Twenty-four single gene interactions were found with *ATPalpha*^*CJ10*^, all but one of which showed suppression of the mutant phenotypes. In total, 35 single gene interactions were found and, importantly, 14 different genes had effects with more than one *ATPalpha* allele (Table 5).

**Table 5:** **Single gene effects confirmed for** ***ATPalpha*** **alleles using classical mutants**

**Cytological region**	**Gene**	**Genotype**	**Putative function** ^**#**^	**ATPα Allele**	**Nature of interaction**	**Significance**
10B3	l(1)10Bb	E04588	Spliceosome component [44]	CJ10	Suppressor	*
21B1-21B1	Galectin	DG25505	Cell surface protein, galactoside binding [45]	DTS1	Enhancer	***
23C9-23C9	Rbp9	∆1	RNA binding [46]	DTS1	Enhancer	*
23C9-23C9	Rbp9	∆1	"	CJ5	Suppressor	****
27E-28B1	Ndae1	MB05294	Sodium driven anion exchanger [47]	CJ5	Suppressor	*
27E-28B1	Ndae1	MB05294	"	DTS1	Enhancer	*
27E-28B1	Ndae1	MB05294	"	CJ10	Suppressor	*
29B4-29E4	Sema-1a	K13702	Axon guidance signal and receptor [48,49]	DTS1	Enhancer	*
29B4-29E4	Glt	EY22126	Cell surface glycoprotein [50]	DTS1	Enhancer	****
29B4-29E4	Glt	EY22126	“	CJ10	Suppressor	*
30C7-30 F2	Nckx30C	E00401	Sodium/Calcium/Potassium exchanger [51]	CJ10	Enhancer	*
30C7-30 F2	Ppk11	MB02012	Excitatory sodium channel [52]	CJ10	Suppressor	***
30C7-30 F2	Ppk11	MB02012	“	DTS1	Suppressor	****
30C7-30 F2	Ppk11	MB02012	“	CJ5	Suppressor	****
30C7-30 F2	nAChRα6	MB06675	ACh receptor subunit	CJ10	Suppressor	*
30C7-30 F2	nAChRα6	MB06675	“	CJ5	Suppressor	****
31C-32E	FKBP59	EY03538	Calcium channel regulator [53]	DTS1	Enhancer	*
31C-32E	FKBP59	EY03538	"	CJ5	Suppressor	***
31C-32E	FKBP59	EY03538	“	CJ10	Suppressor	***
33A8-33B1	Pde1c	C04487	cAMP/cGMP phosphodiesterase [54]	CJ5	Suppressor	****
34E4-35B4	Dyrk2	1	Serine/Threonine kinase [55]	DTS1	Enhancer	***
34E4-35B4	Dyrk2	1	"	CJ10	Suppressor	****
34E4-35B4	Nimb5	MI01793	Bacterial defense	CJ10	Suppressor	**
34E4-35B4	nAChRα5	MB11647	ACh Receptor subunit	CJ10	Suppressor	*
35 F1-36A1	Cact	7	Inhibitor of NF-κB [56]	CJ10	Suppressor	****
36A8-36 F1	Beat-Ia & Fas3	3/E25	Neuronal immunoglobulin-like proteins	CJ5	Suppressor	*
25 F1-36A1	CG5888	MB00188	Toll 3 like receptor	DTS1	Enhancer	****
25 F1-36A1	CG5888	MB00188	"	CJ5	Suppressor	****
46 F1-47A9	CG42732	MB04544	Predicted potassium channel	DTS1	Enhancer	****
46 F1-47A9	Rpl41/NaCP60E	EP348	Ribosomal protein; voltage-gated Na^+^ channel [57]	CJ10	Suppressor	*
46 F1-47A9	CG42732	MB04544	Predicted potassium channel	CJ5	Suppressor	**
46 F1-47A9	Gαo	MI00833	Heterotrimeric G-protein subunit	CJ10	Suppressor	****
46 F1-47A9	CYP49A1 & Gαo	MB04922	Cytochrome P450 & heterotrimeric G-protein subunit	DTS1	Enhancer	****
50B1	CG33156	MB05931	Predicted NAD^+^ kinase	DTS1	Enhancer	****
57C5-57 F6	Pu	r1	GTP cyclohydrolase [58]	CJ5	Suppressor	**
57C5-57 F6	Pu	r1	“	CJ10	Suppressor	****
60E6-60E11	Pain	EP2451	TRP calcium channel [59]	DTS1	Enhancer	**
60E6-60E11	Pain	EP2451	“	CJ10	Suppressor	****
60E6-60E11	Rpl19	K03704	Ribosomal component [60]	DTS1	Enhancer	****
62E8-63B6	Spz5	E03444	Neurotrophin [61,62]	DTS1	Enhancer	**
62E8-63B6	Spz5	E03444	“	CJ10	Suppressor	****
62E8-63B6	Aly	1	Regulator of transcription [63,64]	DTS1	Enhancer	****
62E8-63B6	Rasp	m47	Palmitoyl transferase [65,66]	DTS1	Enhancer	**
62E8-63B6	Rasp	m47	“	CJ10	Suppressor	****
63A3-63A3	Scramb2	EY01180	Predicted phosphatidyl serine scramblase	DTS1	Enhancer	****
63A3-63A3	Scramb2	EY01180	"	CJ10	Suppressor	****
67A2-67D13	Aay	S042314	Predicted Phosphoserine phosphatase	CJ10	Suppressor	**
93B9-93D4	Slmb	295	Ubiquitin ligase [67,68]	DTS1	Enhancer	**
93B9-93D4	Slmb	295	“	CJ10	Suppressor	****
93B9-93D4	Sec15	2	Protein trafficking [69,70]	DTS1	Enhancer	**
93B9-93D4	Sec15	2	“	CJ10	Suppressor	****
93B9-93D4	RhoGAP93B	EY07136	Rac1 GAP [71]	DTS1	Enhancer	*
98 F10-99B9	CG14508	G9163	Predicted cytochrome C	DTS1	Enhancer	***
98 F10-99B9	CG14508	G9163	Predicted cytochrome C	CJ10	Suppressor	****
99E1-3Rt	Sro	1	Ecdysone biosynthetic pathway	CJ10	Suppressor	*

Many genes had an interaction with more than one allele, although some appear to be allele specific. Double mutants were compared to *ATPalpha* ^*^ */+* and heterozygous classical mutant controls. ^*^p < 0.05, ^**^p < 0.01, ^***^p < 0.001, ^****^p < 0.0001.

^#^Function per flybase.org and / or listed citation.

Gal4 driven RNAi strains result in a loss-of-function phenotype and are well-suited to confirm the hypomorphic effect of a heterozygous *Df*. RNAi knockdown was driven with *da-Gal4* in *ATPalpha* mutant backgrounds. *Daughterless* transcripts are stably expressed throughout the life of a fly and are detectable in every tissue by the FlyAtlas affymetrix array analysis [72,73]. We used this driver to ubiquitously express the RNAi constructs and mimic the effect observed with the *Df*. RNAi mediated knockdown of candidate genes was compared to age matched controls lacking the *UAS-RNAi* construct. Twenty-five different genes showed interactions using this method, including 10 genes already identified in the classical mutant screen. Fourteen interactions were identified with *ATPalpha*^*DTS1*^, with nine enhancers and five suppressors. Seventeen interactions, with two enhancers and 15 suppressors, were identified for *ATPalpha*^*CJ5*^. Thirteen interactions, all suppressors, were confirmed with *ATPalpha*^*CJ10*^. In total 15 different genes showed a genetic interaction with two or more *ATPalpha* alleles (Table 6). In total we have identified 50 genes that interact with *ATPalpha*, 25 of which were confirmed to interact with at least two independent alleles.

**Table 6:** **Single gene effects confirmed for** ***ATPalpha*** **alleles using RNAi**

**Cytological region**	**Gene**	**Putative function** ^**#**^	**ATPα Allele**	**Nature of interaction**	**Significance**
21A1-21B1	Galectin	Galactoside binding [45]	CJ10	Suppressor	**
21A1-21B1	Galectin	“	CJ5	Suppressor	***
22 F4-22 F4	CG3528	Unknown	DTS1	Enhancer	*
22 F4-22 F4	CG3528		CJ10	Suppressor	*
22 F4-22 F4	CG3528		CJ5	Suppressor	*
27E-28B1	Ndae1	Na + driven anion exchanger [47]	CJ5	Enhancer	*
29B4-29E4	Glt	Cell surface glycoprotein [50]	CJ10	Suppressor	*
30C8-30C9	Ppk11	Sodium channel [52]	CJ5	Suppressor	**
31C-32E	FKBP59	Calcium channel regulator [53]	CJ5	Suppressor	***
31C-32E	FKBP59	“	DTS1	Enhancer	*
33A1-33A1	Vha100-5	ATPase, proton transport	DTS1	Enhancer	*
33A2-33A2	Esc	Histone methyltransferase component [74]	DTS1	Enhancer	***
33A2-33A2	Esc	“	CJ10	Suppressor	**
34E4-35B4	Dyrk2	Serine/Threonine kinase [55]	DTS1	Enhancer	*
34E4-35B4	Dyrk2	“	CJ5	Suppressor	***
37A2-37A4	Ham	Transcription factor [75]	DTS1	Suppressor	*
37A2-37A4	Ham	“	CJ5	Suppressor	**
37C1-37C1	Ddc	Amino acid decarboxylase [76]	CJ5	Suppressor	***
25 F1-36A1	CG5888	Toll 3 like Receptor	CJ10	Suppressor	**
25 F1-36A1	CG5888	“	CJ5	Suppressor	*
50C5-50C6	Stj	Voltage-gated calcium channel regulatory subunit [77,78]	DTS1	Enhancer	****
50C5-50C6	Stj	“	CJ5	Enhancer	**
51D1-51D1	Cyp6a19	Cytochrome P450	CJ10	Suppressor	*
62E8-63B6	Spz5	Neurotrophin [61,62]	DTS1	Suppressor	**
62E8-63B6	Spz5	“	CJ10	Suppressor	*
62E8-63B6	Rasp	Palmitoyl transferase [65,66]	CJ5	Suppressor	***
63A3-63A3	FMRFaR	Neuropeptide receptor [79]	DTS1	Enhancer	**
63A3-63A3	FMRFaR	“	CJ10	Suppressor	**
63A3-63A3	FMRFaR	“	CJ5	Suppressor	****
64C2-64C5	Con	Homophilic cell adhesion [80]	DTS1	Suppressor	*
64C2-64C5	Con	“	CJ5	Suppressor	**
67A2-67D13	Aay	Predicted phosphoserine phosphatase	CJ10	Suppressor	*
67A2-67D13	Aay	“	CJ5	Suppressor	**
67B9-67B9	Uch-L5	26S Proteasome component [81]	DTS1	Enhancer	*
67D11-67D11	Scramb1	Phosphatidyl serine scramblase	CJ10	Suppressor	**
99B5-99B6	Dop1R2	Dopamine 1-like receptor [82,83]	CJ5	Suppressor	***
99B6-99B6	Ppk21	Sodium channel	DTS1	Suppressor	**
99B6-99B6	Ppk21	“	CJ10	Suppressor	*
100B9-100B9	Ppk24	Sodium channel	DTS1	Suppressor	**
100B9-100B9	Ppk24	“	CJ5	Suppressor	*
100B9-100B9	Ppk24	“	CJ10	Suppressor	**
100C1-100C1	CG11340	Predicted chloride channel	DTS1	Suppressor	*
100C1-100C1	CG11340	“	CJ5	Suppressor	***
100C1-100C1	CG11340	“	CJ10	Suppressor	*

Many genes had an interaction with more than one allele, although some appear to be allele specific. RNAi knockdowns were compared with ATPalpha*, daGal4/+ controls. ^*^p < 0.05, ^**^p < 0.01, ^***^p < 0.001, ^****^p < 0.0001.

^#^Function per flybase.org and/or listed citation.

## Discussion

**Figure 3:**
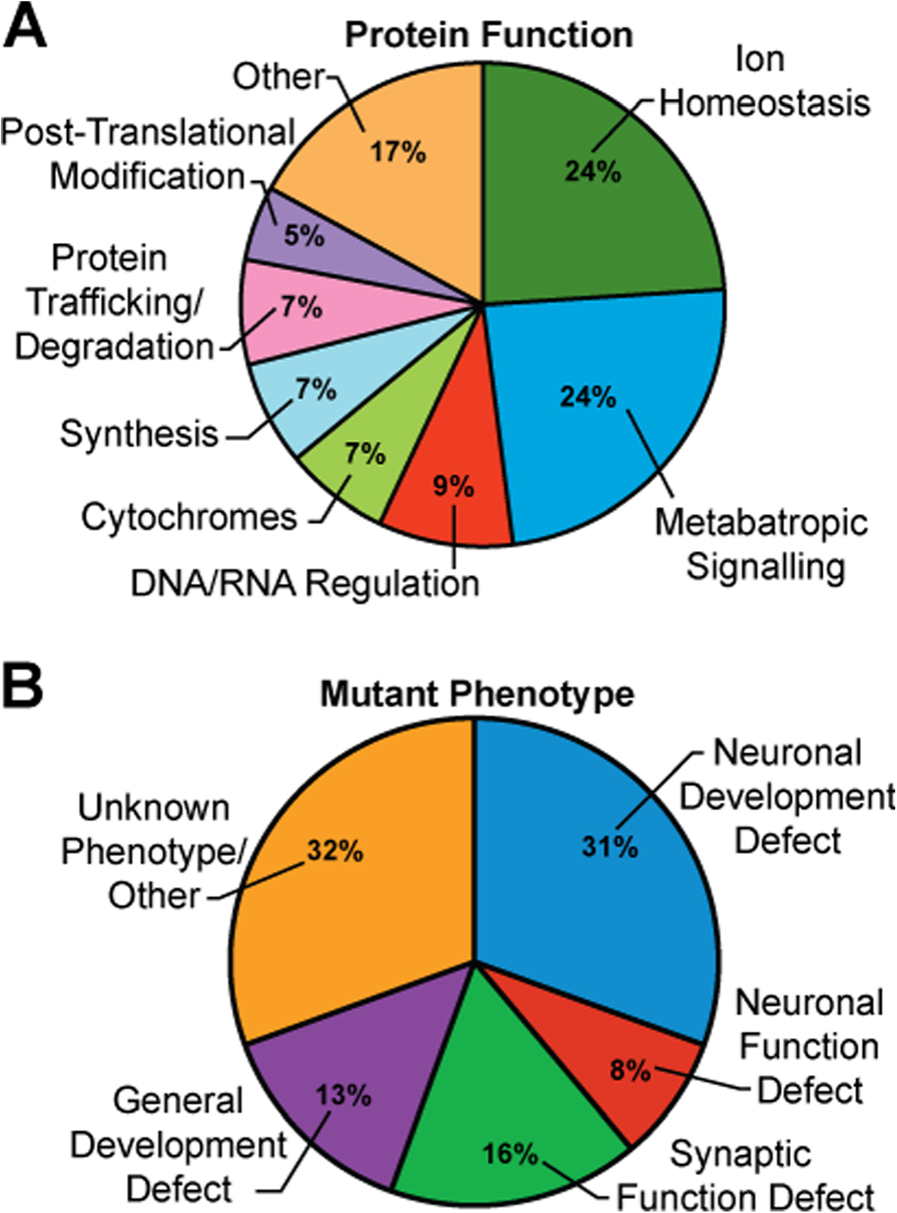
**Distribution of validated genetic modifiers. A**. Protein function of modifiers, as annotated on flybase.org, grouped into major categories. *Stj*, *rasp*, *slmb*, Rpl41/NaCP60E, and *punch* were included in two categories. **B**. Modifier loci categorized according to mutant phenotypes (when available). *FKBP59*, *Cact*, *Scramb1*, and *Stj* were associated with two phenotypic categories.

**Figure 4:**
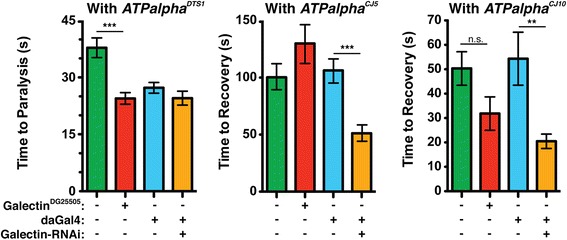
**Genetic interaction between** ***Galectin*** **and** ***ATPalpha*** **.** *Galectin; ATPalpha* double mutants and ATPalpha*, *Galectin* RNAi flies for each *ATPalpha* mutant were assayed and compared to ATPalpha* heterozygous controls. The RNAi knockdown was driven ubiquitously with *daughterless*-Gal4 (daGAL4). The genotypes in each graph are: ATPalpha*/+ (green), *Galectin* ^*DG25505*^ /+;ATPalpha*/+ (red), daGal4,ATPalpha*/+ (blue), and Galectin-RNAi/+;daGal4,ATPalpha*/+ (orange). *Galectin* mutants significantly enhanced the *ATPalpha* ^*DTS1*^ phenotype while *galectin-RNAi* significantly suppress *ATPalpha* ^*CJ5*^ and *ATPalpha* ^*CJ10*^ phenotypes. *p < 0.05, **p < 0.01, ***p < 0.001.

The Na^+^/K^+^ATPase is central to maintaining cytosolic ion homeostasis suggesting that many of the genes identified in our screen would encode proteins that affect cytosolic ion concentrations and, indeed, this was the case (Figure 3A). Nearly 25% of the genes we identified encode proteins with a known function in ion transport. In our search for single gene modifiers we selected genes known to be expressed in the nervous system. Unsurprisingly, ~50% of our hits are known to cause some neuronal defect when knocked out (Figure 3B). For example, most of the cell adhesion and paracrine signaling molecules we found, such as *Galectin* (Tables 5 and 6, Figure 4), *Glt* (Tables 5 and 6), and *Sema-1a* (Table 5) were previously known to cause malformed or improperly targeted synapses [45,48-50]. However, about half of our genes were not previously linked to neuronal function. Additionally, many genes we identified encode proteins implicated in signaling pathways. In particular we found proteins involved in developmental signaling pathways, such as *Wingless* and *Hedgehog* (*rasp* (Tables 5 and 6) and *slmb* (Table 5)), and neuronal growth and survival pathways (*spz5* (Tables 5 and 6)).

**Figure 5:**
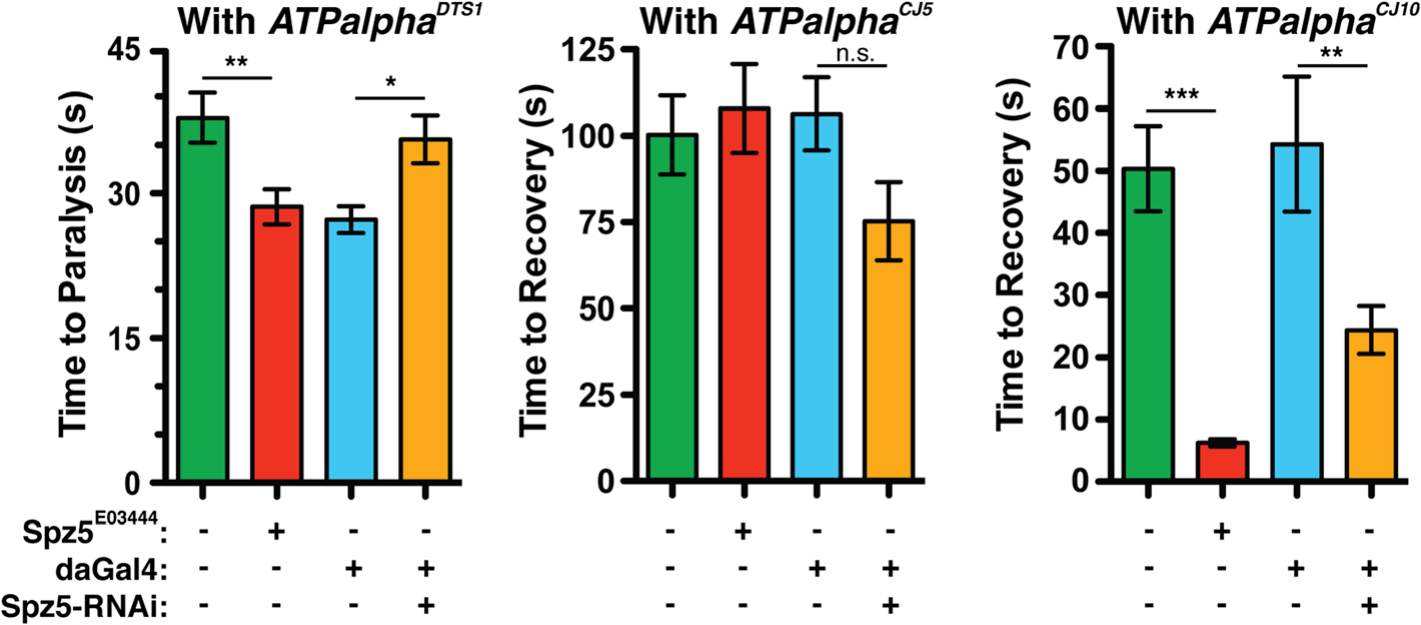
**Genetic interaction between** ***Spz5*** **and** ***ATPalpha*** **.** *ATPalpha/Spz5* double mutants and ATPalpha*, *Spz5* RNAi flies for each *ATPalpha* mutant were assayed and compared to ATPalpha* heterozygous controls. The RNAi knockdown was driven with da-Gal4. The genotypes in each graph are: ATPalpha*/+ (green), *Spz5* ^*E03444*^ /ATPalpha* (red), daGal4,ATPalpha*/+ (blue), and Spz5-RNAi/daGal4,ATPalpha* (orange). *Spz5* mutants significantly enhanced the *ATPalpha* ^*DTS1*^ phenotype but *Spz5* RNAI significantly suppresses the *ATPalpha* ^*DTS1*^ phenotype. The *ATPalpha* ^*CJ10*^ phenotype is suppressed in both the *Spz5* mutant and RNAi. The *ATPalpha* ^*CJ5*^ phenotype was not significantly affected by loss of *Spz5*. *p < 0.05, **p < 0.01, ***p < 0.001.

Spz5 (Figure 5) is especially interesting because it has recently been identified as a *Drosophila* neurotrophin that signals through a Toll receptor [61,62]. Both Slmb and Cact (Table 5) were also identified by our screen and both may function downstream of Spz5. In mammals and flies, Toll signaling activates NF-κB transcription factors, typically through the degradation of an inhibitor of NF-κB (I-κB), such as Cact. Phosphorylated I-κB is targeted for degradation, allowing NF-κB-like transcription factors to translocate to the nucleus. Slmb and its mammalian homolog β-TrCP regulate phospho-I-κB. β-TrCP, and likely Slmb, target an E3 ubiquitin ligase complex to phospho-I-κB and mediate its degradation via ubiquitin proteasome system [68]. Interestingly, we have also identified Uch-L5 (Table 6) in our screen, a member of the 26S regulatory complex which is likely responsible for the deubiquitylation of proteins as they enter the 26S proteasome [81].

Previously published studies of *Slmb*, and *Spz5* show that they play an important role in neural development. Slmb is involved in pruning dendrites and axons during pupation [84] and Spz5 is a neurotrophic signal and axon guidance cue in the embryonic nervous system [61]. Interestingly, animals heterozygous for a loss of function allele of either gene displayed no phenotype in neurons [61,85]. In contrast, our screen examined heterozygous double mutants and found large effects, suggesting *ATPalpha* mutants are sensitive to otherwise inconsequential changes in neuronal development or another unappreciated function of these proteins. Furthermore, a seemingly insignificant disruption of neuronal survival signals early in development may have dramatic phenotypic effects for *ATPalpha* mutants since heterozygosity of *Slmb*, or *Spz5* suppressed the loss-of-function *ATPalpha* phenotype. Additionally, numerous developmental genes were identified implying that neurodevelopmental changes may profoundly affect Na^+^/K^+^ ATPase function or this is a general and potent mechanism to modulate locomotor function.

**Figure 6:**
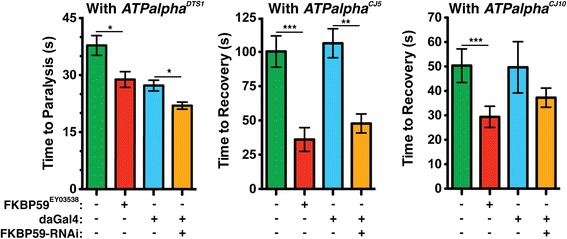
**Genetic interactions between** ***FKBP59*** **and** ***ATPalpha*** **.** *FKBP59; ATPalpha* double mutants and ATPalpha*, FKBP59 RNAi flies were assayed and compared to ATPalpha* heterozygous controls. The RNAi knockdown was driven with da-Gal4. The genotypes in each graph are: ATPalpha*/+ (green), *FKBP59* ^*E03444*^ /+; ATPalpha*/+ (red), daGal4,ATPalpha*/+ (blue), and FKBP59-RNAi/+;daGal4,ATPalpha*/+ (orange). *FKBP59* mutants significantly enhanced the *ATPalpha* ^*DTS1*^ phenotype. The *ATPalpha* ^*CJ5*^ phenotype is suppressed by both the *FKBP59* mutant and RNAi. *p < 0.05, **p < 0.01, ***p < 0.001.

Another interesting possibility is that loss-of-function *ATPalpha* mutations are disrupting neuronal development through alterations in NF-κB signaling. It has been shown that sub-inhibitory concentrations of ouabain activate NF-κB via an Na^+^/K^+^ ATPase dependent mechanism in rat kidney cells. The effect is mediated by slow, inositol triphosphate-dependent, calcium oscillations likely caused by shifting electrochemical gradients [86]. More recently, agrin, a protein involved in synapse formation at NMJs and in the CNS, has been shown to bind to and inhibit the mammalian Na^+^/K^+^ ATPase α3 isoform. Furthermore, agrin seems to bind at the same site as ouabain because a protein fragment can prevent ouabain inhibition of the Na^+^/K^+^ ATPase [87]. Thus it is possible that agrin exerts its effects through NF-κB. If a similar pathway exists in flies it would likely be constitutively active in our loss-of-function mutants and its dysregulation could cause developmental changes, which might increase seizure susceptibility. This is consistent with our finding that knockdown of proteins required for NF-κB activation suppresses seizures in our loss-of-function mutants. NF-κB activation may be caused by calcium oscillations [86], making it possible that some of the calcium channels we found also play a role in this pathway. FKBP59 (Figure 6) is particularly interesting because it inhibits an inositol triphosphate sensitive, non-specific calcium channel, TrpL [53]. Inhibition of calcium channels would likely be required in calcium oscillations. The preponderance of hits related to the NF-κB pathway suggests a possible role for this pathway in seizure pathogenesis.

In most cases the *ATPalpha*^*CJ5*^ and *ATPalpha*^*CJ10*^ mutant phenotypes were modified in the same direction (enhancement or suppression) and they never had opposite phenotypes in our screen. This is consistent with the finding that both exhibit loss-of-function characteristics. The *ATPalpha*^*DTS1*^ phenotype, however, usually contrasted with the phenotypes of *ATPalpha*^*CJ5*^ and *ATPalpha*^*CJ10*^. This is intriguing as *ATPalpha*^*DTS1*^ is a gain-of-function mutation that can be reverted by a second site mutation to give the characteristic *ATPalpha* loss-of-function phenotype [23]. In accord with this fact the vast majority (~ 80%) of the single gene interactions with *ATPalpha*^*DTS1*^ modified the loss-of-function alleles in the opposite direction or not at all. Reduction of Ppk11, Ppk21, and Ppk24 function all suppressed the phenotypes of *ATPalpha*^*DTS1*^ and another allele. All three are predicted epithelial sodium channels (DEG/eNaCs) that function in nociception, mechanosensation, gustation and other sensory functions (Reviewed in [88] and [89]). Thus, it is possible that altered sensory function may underlie the *ATPalpha*^*DTS1*^ paralysis phenotype and that a reduction in the ability of the double mutant animals to sense the elevated temperature is sufficient to suppress the TS paralysis. This possibility is consistent with the kinetics of recovery after animals are returned to the permissive temperature. This is also intriguing as the locomotor dysfunction resulting in hemiparalysis in FHM patients has been reported to be associated with sensory dysfunction and FHM patients report having prolonged visual auras [90-92].

## Conclusions

FHM, RDP, and AHC are complex human neurological diseases associated with mutations affecting the catalytic alpha subunit of the Na^+^/K^+^ ATPase [4-6]. Currently, there is no cure or effective treatment for these diseases. Using three *Drosophila* strains with different missense mutations in *ATPalpha* we have performed a large-scale deficiency screen to identify novel genes that interact with the gene encoding the Na^+^/K^+^ATPase alpha subunit. In total, we have identified 50 genes that interact with *ATPalpha*, 25 of which were demonstrated to interact with at least two independent alleles. We have also implicated 50 critical intervals/deficiency regions for which we have yet to identify individual genes that interact with *ATPalpha* (Tables 2, 3 and 4). Modifier loci that encode proteins expressed in the adult, especially those that phenotypically suppress *ATPalpha* dysfunction, provide proteins/pathways that could be viable targets for the development of new migraine or anti-epileptic drugs. Additionally, studies of these loci and how they modify ATPalpha dysfunction will help us understand epilepsy, hemiplegia and migraine disease pathogenesis in animals.

## Materials and methods

### *Drosophila* strains

Flies were maintained on standard cornmeal-molasses agar medium at 21-22°C. Chromosomal deficiencies were obtained from the Bloomington Deficiency Kit from the Bloomington Stock Center (order date January 2010). The *Df* Kit we received contained 467 stocks with deletions spanning 97.8% of the *Drosophila* genome. Three Na^+^/K^+^ ATPase alpha subunit mutants were used: *ATPalpha*^*DTS1*^ [23], *ATPalpha*^*CJ5*^ and *ATPalpha*^*CJ10*^ [36]. The other *Drosophila* strains used were obtained from the Vienna Drosophila RNAi Center (VDRC) or Bloomington Stock Center.

### Locomotor assays

F_1_ offspring heterozygous for an *ATPalpha* allele and each individual *Df* were collected upon eclosion (day 0) and aged at 25°C on cornmeal-molasses medium. Temperature sensitivity (TS) was assayed on day 1 and bang sensitivity (BS) was assayed on day 15 as described previously [23]. Aged flies were moved to an empty vial in groups of 5 or fewer using an aspirator. For TS, the vial was submerged in a water bath at 38°C such that the flies were restricted to space in the vial below the waterline. A timer was started when the vial was submerged and time to paralysis was recorded for each fly. For BS, the vial was mechanically shaken using a standard lab Vortex Genie 2 (Daigger, IL) on the highest setting for 20 seconds. Time to recovery for each fly was recorded. Both conditional locomotor assays were stopped after 300 seconds.

### *Df* Interaction screen

#### Initial Screens

Males with autosomal deficiencies were mated to *ATPalpha*^*DTS1*^, *ATPalpha*^*CJ5*^, and *ATPalpha*^*CJ10*^ virgin females, and X-linked deficiency virgin females were mated with *ATPalpha*^*DTS1*^, *ATPalpha*^*CJ5*^, and *ATPalpha*^*CJ10*^ males. F_1_ progeny representing a total of 386 deficiency interactions were tested with *ATPalpha*^*DTS1*^ animals (83% of *Df* kit), 393 were tested with *ATPalpha*^*CJ5*^ (84% of *Df* kit), and 358 were tested with *ATPalpha*^*CJ10*^ animals (77% of *Df* kit). Each of the 467 *Dfs* we received was tested with at least one *ATPalpha* allele, the vast majority were tested with multiple alleles and >55% were tested with all three alleles. Assays were performed as described above.

#### Verification screen

Putative modifier *Df* strains identified in the initial screen were retested in an independent experiment to verify the findings and reduce the rate of false positives. In selecting *Df* stains to test again, we favored *Dfs* that suppressed *ATPalpha* mutant phenotypes and/or interacted with more than one *ATPalpha* allele. During the verification screen all three *ATPalpha* alleles were investigated.

### Single gene identification

We developed an analysis called the Reproducibility Index (RI) in order to guide our search for single gene modifiers of the *ATPalpha* alleles. The goal of this index was to rank the most promising *Df* intervals based on the magnitude and reproducibility with which they modified an *ATPalpha* allele phenotype. To this end, we first calculated the number of standard deviations of the Df, ATPalpha* double mutant mean from the total mean of the primary screen of each *ATPalpha mutant* using:

$$ Num.Std.Dev.\left(\#SD\right)=\frac{Mea{n}_{total}-Mea{n}_{Df}}{StdDe{v}_{total}} $$

where StdDev_total_ is the standard deviation of all deficiencies in the primary screen, Mean_total_ is the mean of all deficiencies in the primary screen, and Mean_Df_ is the mean response of a *Df/ATPalpha* double mutant. Num.Std.Dev (#SD) was calculated for the mean response of a *Df* double mutant in the primary (#SD_prim_) and verification (#SD_veri_) screen. We reasoned that these values provide a normalized metric of how much a *Df* modified an *ATPalpha* phenotype in each trial. We used these values to calculate the RI:

$$ RI=\left|\operatorname{}S{D}_{prim}+\#S{D}_{veri}\right|-AV/2 $$

where

$$ Absolute\  Varience(AV)=\left|\#S{D}_{prim}-\#S{D}_{veri}\right| $$

The RI increases for *Dfs* that were further from the total mean and decreases for *Dfs* that varied more across the two trials. Thus, a high RI suggests that a region is more likely to contain a gene that interacts with and modifies an *ATPalpha* allele in a reproducible manner. In some intervals we were able to use small *Dfs* to narrow the interval further. We, again, prioritized strongly suppressing intervals over enhancing intervals and intervals that interacted with multiple alleles. Single genes were selected from critical intervals using the G-Browse feature (an annotated genome) of flybase.org. In some very small intervals all genes in the region were tested. In large intervals we necessarily focused on genes with described expression within the nervous and or muscular systems, introducing a noted bias. Many of the alleles chosen were P-element or classical mutations reported to knockout the genes of interest. The stocks of interest were ordered from the Bloomington Stock Center.

### RNAi analysis

When classical mutants were unavailable for certain loci or to confirm an interaction found using a classical mutant, RNAi analysis was used to examine the gene in question. RNAi stocks were ordered from the VDRC. The RNAi transgenes were driven using *daughterless* Gal4 strains (*daGal4*) in each *ATPalpha* mutant background. RNAi male flies were mated to ATP*alpha*, *daGal4* virgin females. Progeny were raised at 25°C, and TS and BS tests were performed as described previously.

### Data collection and statistics

Data were collected and organized using Microsoft Excel (Redmond, WA). Data were analyzed in GraphPad Prism 5 (San Diego, CA). We used ANOVA to compare the *ATPalpha* mutant heterozygotes, the classical mutant heterozygotes, and flies heterozygous for both alleles. Tukey’s multiple comparison test was performed to determine if the double mutants were significantly different from the *ATPalpha* mutant heterozygote and the classical mutant heterozygote. Adjusted p-values are reported in Table 5. The effect of RNAi transgenes was analyzed using a Student’s *t-test* to determine if single gene knockdowns significantly modified the phenotype of ATPalpha*, daGal4 controls. Significant interactions are reported in Table 6.

## Additional file

Additional file 1:
**Data from the primary and verification** ***Df*** **screens.**
